# Self-reported measures in health research for people with intellectual disabilities: an inclusive pilot study on suitability and reliability

**DOI:** 10.1186/s12874-018-0539-1

**Published:** 2018-07-16

**Authors:** Kristel Vlot-van Anrooij, Hilde Tobi, Thessa I. M. Hilgenkamp, Geraline L. Leusink, Jenneken Naaldenberg

**Affiliations:** 1 Department of Primary and Community Care, Radboud University Medical Center, P.O. Box 9101, route 68, 6500 HB Nijmegen, The Netherlands; 2 Biometris, Wageningen University, Wageningen, The Netherlands; 3 Intellectual Disability Medicine, Department of General Practice, Erasmus Medical Center, Rotterdam, The Netherlands

**Keywords:** Intellectual disability, Self-report, Surveys and questionnaires, Test-retest reliability, Methodology, Physical activity, Sedentary behaviour, Self-reported health, Inclusive research

## Abstract

**Background:**

The lack of suitable and reliable scales to measure self-reported health and health behaviour among people with intellectual disabilities (ID) is an important methodological challenge in health research. This study, which was undertaken together with co-researchers with ID, explores possibilities for self-reported health scales by adjusting, testing, and reflecting on three self-reported health scales.

**Methods:**

In an inclusive process, the researchers and co-researchers with ID adjusted the SBQ (sedentary behaviour), SQUASH (physical activity), and SRH (self-reported health) scales, after which a test-retest study among adults with ID was performed. Test outcomes were analysed on suitability and test-retest reliability, and discussed with the co-researchers with ID to reflect on outcomes and to make further recommendations.

**Results:**

Main adjustments made to the scales included: use easy words, short sentences, and easy answer formats. Suitability (*N* = 40) and test-retest reliability (*N* = 15) was higher for the adjusted SQUASH (SQUASH-ID), in which less precise time-based judgements are sought, than in the adjusted SBQ (SBQ-ID). Suitability and test-retest reliability were *fair to moderate* for the SRH-ID and CHS-ID. The main outcome from the reflection was the recommendation to use SQUASH-ID answer options, in which less precise time-based judgements were sought, in the SBQ-ID as well.

**Conclusions:**

This study served as a pilot of an inclusive process in which people with ID collaborated in adjusting, testing, and reflecting on self-reported health scales. Although the adjusted self-reported measurements may be reliable and suitable to the target group, the adjustments needed may impair measurement precision. This study’s results contribute to informed decision making on the adaptation and use of self-reported health scales for people with ID.

## Background

In the current patient-centred paradigm, self-reports such as patient-reported outcome measures and health behaviour are highly valued in care and research. Self-reports, often collected via questionnaires, can help to make shared decisions and tailor treatment plans [1, 2]. Socially disadvantaged groups, such as people with intellectual disabilities (ID), who have impaired social functioning and limited cognitive ability that developed before the age of 18 [3], are likely to be underrepresented in self-report studies [4–6] because of challenges in all steps of research: (1) sampling; (2) recruitment and gaining consent; (3) data collection and measurement; (4) intervention, delivery, and uptake; and (5) retention and attrition [7]. The present paper focuses on data collection and measurement.

In data collection on health behaviour and patient-reported outcome measures for people with ID, questionnaires are often proxy-administered [8–11]. It may be difficult to find good proxy respondents who have a high level of interaction with the person with ID, have known the person for a long time, and relate to the type of domain being queried [12]. Also, providing high quality answers can be difficult for proxy respondents, as shown in a study by Andresen and colleagues where proxies tended to overestimate impairment and underestimate health-related quality of life [13]. Besides the challenges of proxy-administered questionnaires [12], the need to listen to the views and experiences of people with ID themselves is increasingly acknowledged [14].

Self-reports of people with ID potentially contribute to the improvement of healthcare research for this group and the autonomy of people with ID, and answer their wish and democratic right to be involved in research [15–17]. Furthermore, self-reported instruments may contribute to the growing demand for inclusive research as required by funding bodies and national policies [18–20]. Despite the fact that the active involvement of people with ID in health research, either as respondents or as part of a research team [16], is increasingly popular [18, 20, 21], suitable and valid scales to collect self-reports on health and health-related behaviour among people with ID remain to be scarce [10, 22]. Online questionnaires which allow for data collection among large samples are required as the field of research for people with ID is in need of studies with larger sample sizes. Also, compared with interviews, questionnaires are less prone to acquiescence, social desirability and interviewer effect, which are important methodological challenges in data collection among people with ID [10, 22, 23]. So, adjusted versions of questionnaire scales designed for the general population are needed to tackle the methodological challenges of data collection among people with ID [10, 12, 22]. This pilot study aims to explore the applicability of self-reported health scales in research among people with mild ID, by adjusting, testing, and reflecting on three self-reported health scales together with co-researchers with ID.

## Methods

### Study context

Important contextual factors for study designs in which people with ID participate are: access to the population, ethical concerns, and the abilities of the target group [22, 24]. First, poor access to the population is due to: A) a lack of population-based registries of this population [24, 25], and B) organisational barriers to recruitment (e.g. obtaining organisational consent, communication problems, support of employees) when sampling through residential service providers [16, 24]. In this study the opportunity was taken to recruit amongst the large group of Special Olympics participants. Second, the burden and potential benefits for this vulnerable participant group should be carefully considered from an ethical point of view. Both researchers and co-researchers with ID assessed the original self-reported health scales as too difficult. They deemed the administration of these scales a probable cause of unnecessary stress, and, therefore, unethical. Hence, this stresses the demand for adapted versions of the scales. Finally, the following characteristics of people with ID ought be taken into account: A) the heterogeneity of the cognitive and linguistic abilities of people with ID [22]; B) the difficulties that people with ID have in making time-based judgements and comparisons [22]; and C) the high tendency towards acquiescence among people with ID [12].

### Data collection

This inclusive study on self-perceived health and health behaviours amongst people with ID consisted of three phases: (1) adjusting the three health scales; (2) performing an online test-retest study of the adjusted scales among people with ID; and (3) reflecting on the adjusted scales and the test-retest study results. To facilitate an inclusive approach, people with ID participated actively during the study as co-researchers [21] in phases 1 and 3. Five co-researchers who had been involved in previous studies by our research group were invited to participate in this research project because of their experience in advising on data collection.

### Phase 1: inclusively adjusting the health scales

Three health scales frequently used in the general population were selected by the researchers. Adjustments to the scales, the informed consent procedure, and the outline of the online questionnaire for people with ID were discussed by two co-researchers and the principal researcher, resulting in a list of recommendations according to which the researchers adjusted the questionnaire. Then, the adjusted questionnaire was pilot tested by three other co-researchers. Their feedback, together with recommendations from relevant literature [10, 12, 22, 23, 26], was used by the researchers to develop the final questionnaire. The recommendations and the adjusted scales are described in the results section.

### Phase 2: test-retest of the adjusted scales

#### Sampling, recruitment, and informed consent

To test the adjusted scales among adults with mild ID, all adult participants in the Three Day March, part of the Dutch Special Olympics 2016, were invited to participate. The register for the Three Day March included email addresses for the participants’ support person only. These support persons, often a family member or professional caregiver, received an invitation by email giving information on the study. They were asked to discuss participation in the study with the person(s) they supported and to discuss whether this person met the inclusion criteria of: having intellectual disabilities, being adult, being able to give informed consent, and being able to answers questions. When a support person served a group of up to five persons, a personalised invitation was sent for each person with ID. Support persons serving a group of more than five persons with ID received a general invitation followed by a phone call from the first author.

Risks of, and objections to, participation were deemed to be negligible in our study, which asks respondents to fill out a questionnaire on health-related behaviour and self-reported health. Potential respondents with sufficient decision capacity according to their support persons were asked to give informed consent, as suggested by Iacono and Murray [27]. After consent was expressed to the support person, the potential participants opened the online questionnaire. The first part of the questionnaire contained study information and concluded with three questions to check whether the respondent understood the study information and the informed consent procedure. Thereafter, informed consent was obtained online. At the end of the questionnaire, respondents were invited to participate in the retest, 2 weeks later.

#### Measurements

The original scales are the Sedentary Behaviour Questionnaire (SBQ), Short QUestionnaire to ASsess Health-enhancing physical activity (SQUASH), and a single-item scale on self-reported health (SRH). These scales are often used in health research [28–34]. The original scales are explained below. In the results section, the first phase of questionnaire adjustment, the informed consent procedure, and the adjustments to the three scales are reported.

The SBQ aims to measure the amount of time spent on nine sedentary activities: watching television, playing computer/video games, sitting while listening to music, sitting and talking on the phone, doing paperwork or office work, sitting and reading, playing a musical instrument, doing arts and crafts, and sitting and driving in a car, bus, or train. The question asked in the SBQ is: *‘On a typical weekday/weekend day, how much time do you spend doing the following?’* Answer options are: none, 15 min or less, 30 min, 1 h, 2 h, 3 h, 4 h, 5 h, or 6 h or more. The item, total hours per week spent on sedentary activities, is calculated by multiplying weekday hours by five and the weekend day hours by two and summing these. Total hours spent on sedentary behaviour per day is calculated by dividing total hours per week by seven. Outcomes higher than 24 h per day are usually truncated to 24 h per day [35].

The SQUASH assesses physical activity levels and may be used to measure compliance with physical activity guidelines [36]. It contains questions about the following sets of activities: (A) *commuting activities* (walking to/from work school, bicycling to/from school), (B) *leisure-time activities* (walking, bicycling, gardening, odd jobs, and sports), (C) *household activities* (light household work, intense household work), and (D) *activities at work and school* (light work, intense work). For each activity, questions are asked about the number of *days per week (*open answer box), *average time per day* (open answer box), and *effort* (multiple choice: light, moderate, or intense) involved in the activity [36].

Finally, the question *‘How would you rate your current general health on a scale from 1 to 10? (score 1=very bad, score 10=perfectly healthy)’* aims to measure self-reported health (SRH).

#### Data analysis

The adjusted scales data, obtained in the online test and retest study, were analysed on suitability and reliability. Prior to analysis, data processing included the transformation of strings into numerical variables for the SBQ-ID according to the following rules: (1) answers such as ‘no’ and ‘never’ were given the numeric code ‘0’; (2) for answers containing a range of values, the middle of that range was used, e.g. ‘two–three hours’ yielded 2.5; and (3) soft quantifiers, such as ‘rarely’ and ‘sometimes’, were regarded as non-quantifiable answers. For the test-retest reliability of the SBQ-ID, missing values were coded as 0 h.

Indicators for suitability were response rate and the proportion of non-quantifiable and missing values, respectively. For interval measurements, the test-retest reliability was investigated by means of the Intraclass Correlation Coefficient (ICC) with a 95% confidence interval (CI). For categorical variables, the test-retest reliability was investigated by means of Kappa with a 95% CI calculated using bootstrapping [37]. The ICC and Kappa values were interpreted as follows: 0.00–0.20 as poor, 0.21–0.40 as fair; 0.41–0.60 as moderate; 0.61–0.80 as substantial; and 0.81–1.00 as almost perfect reliability [38]. Convergent validity was estimated through the correlation, Kendall’s tau (*τ),* between the two self-reported health scales (SRH-ID and CHS-ID). The statistical analysis was conducted using SPSS version 22.

### Phase 3: reflecting on adjusted scales and results in group discussion

The results of the test-retest study and the adjusted scales were discussed in two separate group discussions with two and three co-researchers respectively, the principal researcher and a moderator experienced in group discussions with people with ID. A PowerPoint presentation and A3 posters were used to show the participants the adjusted questionnaire and the results of the test-retest study. During the group discussions, the co-researchers reflected for each scale on the adjusted format and the results of the test-retest study and identified recommendations for further improvement. The transcription of the group discussions were thematically analysed [39] on: (1) reflections on adjusted questionnaire, (2) reflections on test-retest results, and (3) recommendations for further improvements to the questionnaire.

## Results

### Phase 1: inclusively adjusting the health scales

The discussion with the co-researchers with ID and the feedback from the pilot yielded the following general recommendations: (1) include questions to check whether the study information and the meaning of an informed consent is understood correctly, (2) group related questions, (3) depict per page or screen questions on one single theme only, and (4) explicitly allow the participant to ask for, and receive, help from a support person. Specific recommendations for the settings and layout of an online questionnaire were: (1) use of a clear font and large font size, (2) allow for item non-response, and (3) use multiple pages because scrolling down requires more motor skills than a single carriage return does. The co-researchers suggested many adjustments tapping clarity of language, such as use of easy words, easy answer formats, and short sentences. The co-researchers were indecisive on whether or not the SBQ and SQUASH, asking for *hours spent on certain activities,* had to be adjusted as the time-based judgement sought might be too in-depth. Hence, the SQUASH question format was altered, whereas the SBQ format was maintained, allowing comparison of suitability of both formats.

In the adjusted SQUASH (SQUASH-ID), the physical activities for which judgements were sought were the same as in the original SQUASH. However, the question format was altered from *days per week*, *average time per day*, and *effort* to *intensity* and *days per week*. For each activity, respondents were asked to report on (1) the *intensity* with which they did this activity by choosing one of the tick box options: never, sometimes, often, or always; and (2) *days per week*, by ticking the days of the week when they normally do this activity (tick box with Monday–Sunday).

For the adjusted SBQ (SBQ-ID), the question phrasing was slightly changed (‘*How many hours are you sitting on a weekday (Monday to Friday) when you are ...?’)* and an example was added. The co-researchers suggested changing the original multiple choice answer categories to an open answer box to allow respondents to express the time verbatim. Weekend days were split into Saturday and Sunday because activities on these days varied a lot according to the co-researchers.

The question *‘What score between 1 and 10 do you give for your current general health? (score 1=very bad, score 10=perfectly healthy)’* was rephrased as: ‘*What score do you give your own health? (score 1=very bad, score 10=perfectly healthy)’.* As recommended by the co-researchers, one other question was added, namely, the health ladder, which has been used previously [40]. The health ladder consisted of the question ‘*How healthy do you feel?’* with the instruction *‘Place the arrow on the health ladder; green is very healthy, red is very unhealthy’*. The colours, or answer categories, on the ladder were green, yellow, light orange, dark orange, and red.

These points were all taken into account in the programming of the questionnaire in Limesurvey [41]. Estimated time to complete the questionnaire was between 15 and 30 min.

### Phase 2: test-retest of the adjusted scales

#### Overall response

**Fig. 1:**
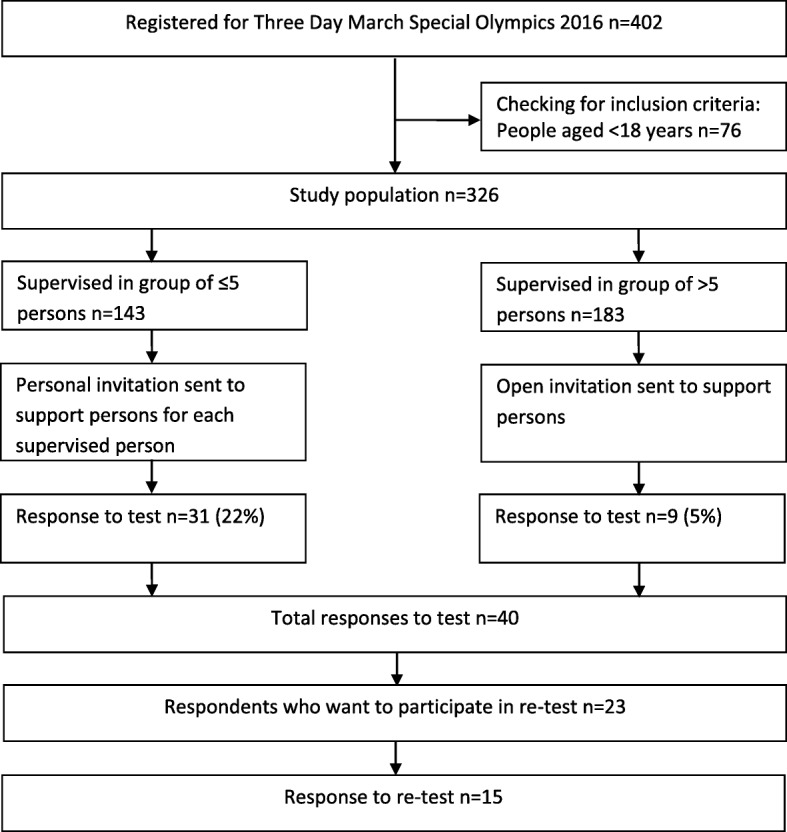
Participation flowchart

To pilot test the SBQ-ID, the SQUASH-ID, the Self-Reported Health scale for people with ID (SRH-ID), and the Coloured Health Scale for people with ID (CHS-ID), people with ID were invited to participate in this study (see Fig. 1). Some support persons who had received a personal invitation explained why they would not participate: the person with ID did not want to (*N* = 16), the person with ID did not meet the inclusion criteria (*N* = 14), or the support person would be absent during the study period (*N* = 2). In total, 40 persons filled out the questionnaire of which 31 with help from someone else. The group consisted of 18 males and 22 females and their age ranged from 18 to 76 (mean = 37, SD = 15.5). Participants lived in a community group home (*N* = 15), independent with ambulatory support (*N* = 10) or with their parents (*N* = 7). For daytime activities most participants reported day-care (*N* = 19), and paid work (*N* = 13), where few reported voluntary work (*N* = 3) or school (*N* = 4). Out of the 40 respondents, 23 were willing to be approached 2 weeks later for the retest. Of these 23 persons, 15 persons answered the questionnaire twice.

#### Sedentary behaviour questionnaire for people with ID (SBQ-ID)

For the SBQ-ID, missing values varied per question, from 2 to 12 out of 40 respondents. The provisions of non-quantifiable values also varied per question, ranging from 3 to 6 out of 40 respondents. These non-quantifiable answers were: 1) *soft quantifiers* such as ‘sometimes’, ‘not much’; 2) *time frames* such as ‘in the morning’, ‘before bedtime’; 3) *conditional answers* such as ‘depends on ….’, ‘varies every day’; 4) *related to the respondents disability* such as ‘wheelchair bound’, ‘I cannot do that’; or 5) *associative answers* such as ‘coffee’ when hours of sitting while eating and drinking was asked.

Due to missing values, the total hours of sedentary activities could be calculated only for 16 respondents and had a median of 10 h per day (IQR 6.00–15.61). One respondent reported a total time spent on sedentary activities per day that exceeded 24 h. The reliability test of the SBQ-ID showed heterogeneous results (Table 1). The item ICC ranged from poor for *Eating or drinking* (0.09) and *Transport* (− 0.14) to substantial for *Playing a musical instrument* (0.79). Because of high numbers of non-quantifiable answers, the summary values *hours of sedentary activity on a weekday/Saturday/Sunday/per week* could be calculated for four to eight respondents: too few to calculate ICC.

**Table 1:** SBQ-ID, Test suitability and Test-retest reliability

Items	Test suitability (*N* = 40)		Test-retest reliability (*N* = 15)	
How many hours are you sitting on a weekday/Saturday/Sunday^a^ when you are....	N non-quantifiable answers	N missing answers	N	ICC (95% CI)
Eating or drinking	3	5	14	0.09 (−0.45;0.56)
Watching TV, movies, or series	3	8	13	0.35 (−0.22;0.74)
Using computer or tablet	3	7	13	0.26 (−0.32;0.69)
Listening to music on radio or CD	4	9	12	0.30 (−0.30;0.73)
Using a telephone	4	8	13	0.36 (−0.22;0.75)
Playing a musical instrument	5	11	12	0.79 (0.42;0.94)
Reading	6	10	13	0.26 (−0.31;0.70)
Travelling	5	9	11	−0.14 (− 0.66;0.48)

^a^This question was asked separately for a weekday, Saturday, and Sunday; values are reported for average per week

#### Short QUestionnaire to ASses Health-enhancing physical activity for people with ID (SQUASH-ID)

For the SQUASH-ID, suitability can be reported only for the *intensity* items because the days per week items had tick boxes as answers option, making it impossible to distinguish missing values from ‘none of these apply’. For the *intensity* items, missing values were low; only 1 out of 40 respondents did not answer the items. As the answer options were closed, there were no non-quantifiable values. The test-retest reliability results are shown in Table 2. For the *days per week* items, 7 out of the 11 SQUASH-ID items showed a substantial to almost perfect correlation. In Table 2, Kappa (95% CI) is also reported. Kappa values (95% CI) for the test-retest of *intensity* were predominantly moderate.

**Table 2:** SQUASH-ID, Test-retest reliability

Items	Test-retest reliability (*N* = 15)		
	N	N of days per week ICC (95% CI)	Intensity Kappa (95% CI)
Commuting activities			
Walking	15	0.92 (0.77;0.97)	0.61 (0.21;1.00)
Biking	15	0.88 (0.67;0.96)	0.40 (0.04;0.72)
Activity at work and school			
Light activities	15	0.27 (− 0.26;0.68)	0.18 (− 0.09;0.48)
Intense activities	15	0.80 (0.51;0.93)	0.45 (0.08;0.77)
Household activities			
Light household activities	15	0.82 (0.54;0.93)	0.45 (0.10;0.78)
Intense household activities	15	0.57 (0.10:0.83)	0.65 (0.30;1.00)
Leisure-time activities			
Walking (leisure time)	15	0.72 (0.48;0.93)	0.50 (0.13;0.81)
Bicycling (leisure time)	15	0.64 (0.21;0.86)	0.17 (−0.22;0.61)
Gardening	15	0.61 (0.16;0.85)	0.55 (0.13;0.88)
Odd jobs	15	0.14 (−0.38;0.60)	0.63 (0.32;1.00)
Sports	15	0.18 (−0.35;0.62)	

#### Self-reported health scale for people with ID (SRH-ID) and coloured health scale for people with ID (CHS-ID)

The two single-item scales on self-reported health from Phase 1 were the SRH-ID requiring a 1–10 score and the CHS-ID requiring a colour score. Thirty-five persons answered the CHS-ID and 36 persons answered the SRH-ID. The median score was *yellow* for the CHS-ID and *8* for the SRH-ID. Although answers provided to the CHS-ID covered all answer options, respondents gave no scores below 5 on the SRH-ID. For both single-item scales, the test-retest reliability was about 0.40. While answers to the CHS-ID scale covered all answer options, on the 1–10 scale respondents did not give a score below 5. The correlation between the CHS-ID and SRH-ID scales was strong (*τ* = 0.73, *P* < 0.001) (Table 3).

**Table 3:** SRH-ID and CHS-ID, Test suitability and Test-retest reliability

Scales	Test suitability (n = 40)	Test-retest reliability (n = 15)	
	n missing answers	n	ICC (95% CI)
SRH-ID	4	15	0.39 (0.14;0.74)
CHS-ID	5	13	0.41 (0.13;0.71)

### Phase 3: reflecting on adjusted scales and results in group discussion

#### Reflections and recommendations for the adjusted scales

In the reflection phase the co-researchers discussed the results of phase 2 and identified possible improvements of the adjusted scales.

In the SBQ-ID many missing and non-quantifiable answers had been reported. Looking at these results the co-researchers believed the question format, in which *hours spent on an activity on a weekday* were queried, to be very difficult. This questions format was deemed to be too difficult because it requires remembering activities over a week’s time, awareness of time, and numeracy skills. Suggested possible improvements include: 1) use the same answer type as used in the SQUASH-ID; 2) structure items in the categories ‘commuting activities’, ‘activity at work, day-care and school’, ‘household activities’ and ‘leisure time activities’; and 3) give examples of the items.

Comparing the results of the SBQ-ID with the SQUASH-ID, the co-researchers valued the SQUASH-ID scale as much easier due to clearer answer options and requiring less detailed time-based judgements (estimating and calculating hours was not needed). Nonetheless, the co-researchers identified some possible difficulties in SQUASH-ID, including understanding what intense activities mean, understanding the difference between leisure time and work, and fitting in activities which are not specifically asked for in the items. Recommendations to improve the SQUASH-ID included: 1) clarify intense activities by listing physically intense activities; 2) change the questions on walking and biking in leisure time slightly, into *‘Do you walk in leisure time, that is not to get to school, work or day care?’; and 3) providing example activities.*

Comparing the results of the CHS-ID and SRH-ID, the co-researchers considered the CHS-ID as easier than the SRH-ID. A suggestion to make the SRH-ID easier was to include a row of numbers or to combine the colour scale with the numbers. Differences between colours on the CHS-ID were unclear for one co-researcher which could be mitigated by the use of more contrasting green and red colours and by placing a line between the colours, or adding numbers. For the SRH-ID, the co-researchers reflected that respondents might not have given answers lower than 5 because a 6 is usually valued as sufficient and below 6 as insufficient and bad.

#### Reflection on test-retest differences

The co-researchers provided possible explanations for the test-retest results. The co-researchers argued that people might have become aware of their own behaviour and therefore gave another answer the second time they answered the questions, which describes a research effect. Co-researchers also suggested changes in health state, leisure activities, or weather conditions, and, forgetting may have caused differences between test and retest answers.

## Discussion

This study aimed to explore possibilities for self-reported health scales by adjusting, testing, and reflecting on self-reported health scales in an inclusive manner. In the adjustment phase, the co-researchers with ID gave recommendations for the online questionnaire in general and specifically for the scales. Please note that the items of the SQUASH and SBQ were used as starting point. Pilot testing the adjusted scales on suitability among 40 persons with ID suggested that the SQUASH-ID was more suitable than the other scales, as non-response was higher in the SBQ-ID, the SRH-ID, and the CHS-ID. Pilot testing the adjusted scales on test-retest reliability among 15 persons with ID showed a test-retest reliability of the items of the three scales, varying between *poor* and *almost perfect*. In the reflection phase, building on the results of phase 2, further recommendations were done. Answer options that require less detailed memories and calculations, like *days per week* and *intensity* as used in the SQUASH-ID, seem to be more suitable to the cognitive abilities of people with mild ID than answer options in the SBQ-ID.

### Inclusively adjusting and reflecting on health scales

By using the described approach, we aimed to gain a better insight into what is needed to design measurement instruments that better fit the capacities of people with ID and how this may be achieved in an inclusive manner. The co-researchers provided a respondents’ perspective by carefully and patiently discussing the scales, which, according to the literature, is a very important issue in adapting measurements for self-reports of people with ID [10, 26]. In the adjustment phase, co-researchers helped to apply general rules stated in the literature on informed consent, questions, easy language and settings and lay out of the questionnaire. [10, 12, 14, 22]. During the reflection phase the adjusted scales and test outcomes provided clues for the in-depth discussion on further recommendations. Concluding, thanks to the inclusive process, the researchers and co-researchers got insights that they might not had gained otherwise.

### Suitability and reliability of the scales

The results from this pilot study indicate that the better a scale is adjusted to the target population, the better the scale performs on suitability. In our study, the SQUASH-ID scale, in which less precise time-based judgements are sought, was more suitable than the SBQ-ID scale. Although caution should be taken when discussing the test-retest results because of the small sample size, it seems the SQUASH-ID scale performs better than the SBQ-ID scale. Although our results suggest that simplification of time-based judgements increases suitability and yields more reliable data, there is a cost also; it affects measurement equivalence to the original scales and reduces the precision of the concepts’ measurement.

In general, it is difficult to develop reliable items and scales to measure time-based judgements of behaviour [42, 43]. The test re-test reliability of the SQUASH-ID and SBQ-ID were somewhat lower than in the studies where the original scales were tested (with *N* = 49 and 50, respectively) [35, 36]. This lower reliability could be partly explained by the fact that behaviour patterns among people with ID are prone to change as a consequence of changed availability of support persons. The item test-retest reliability of the SQUASH-ID and SBQ-ID varied strongly, just like in the original scales. The two versions of the single-item questionnaire for self-reported health (the SRH-ID, and the CHS-ID) correlated strongly with each other, although both showed poor test-retest reliability. Further research on these scales is necessary, including the exploration of the last recommendations of the co-researchers.

#### Strengths and limitations

To the best of our knowledge, this study piloted an inclusive process in which people with ID contributed to the adjustment, testing, and reflecting on the suitability and reliability of self-reported health scales for people with ID. This study suffered from difficulties in recruitment, a commonly mentioned problem in studies among people with ID [24]. Despite the fact that a large sample was invited to participate, only a small group participated in our study. Support persons were gatekeepers to participating in this study, a commonly mentioned problem in studies among people with ID [24]. The retest phase took place over summer a period (holidays) during which, support to fill in the questionnaire can be hampered. The heterogeneity of people with ID with respect to levels of cognitive and linguistic abilities need be taken into account [22]. Our sampling strategy aimed at people with mild ID who are interested in physically activity, which is a selective sample.

This study described a pilot of scale adjustment by means of an inclusive procedure. Further research is needed to test reliability and investigate (face, content, construct, concurrent and predictive) validity of the SBQ-ID, the SQUASH-ID, the CHS-ID, and the SRH-ID in a large and diverse sample of people with ID. Testing responsivity of the scales in a longitudinal study is required to investigate whether these scales could be used in physical activity intervention studies. Although testing the scales in an online questionnaire may be convenient and time saving, testing the scales in a face-to-face mode should also be considered as this might improve response rate and decrease item non-response. In general, to increase the quality and availability of measurement instruments for this population, more projects are needed in which scales are adjusted together with people with ID and tested on reliability and validity.

## Conclusion

This study contributes to informed decision making on using self-reports and adjustments to self-reported health scales for people with ID. This pilot study’s results indicate that commonly used self-reported measurements can be made suitable to people with ID in an inclusive process and may yield reliable scales. Nonetheless, scale adjustment may reduce measurement equivalence with original scales.

## Abbreviations

CHS-ID: Coloured Health Scale for people with ID
CI: Confidence interval
ICC: Intraclass correlation coefficients
ID: Intellectual disabilities
SBQ: Sedentary Behaviour Questionnaire
SBQ-ID: Sedentary Behaviour Questionnaire for people with ID
SQUASH: Short QUestionnaire to ASsess Health-enhancing physical activity
SQUASH-ID: Short QUestionnaire to ASsess Health-enhancing physical activity for people with ID
SRH: Self-reported health scale
SRH-ID: Self-reported health scale for people with ID
